# Extending TCGA queries to automatically identify analogous genomic data from dbGaP

**DOI:** 10.12688/f1000research.9837.1

**Published:** 2017-03-24

**Authors:** Erin K. Wagner, Satyajeet Raje, Liz Amos, Jessica Kurata, Abhijit S. Badve, Yingquan Li, Ben Busby

**Affiliations:** 1 BioStat Solutions, Frederick, USA; 2 National Library of Medicine, National Institutes of Health, Bethesda, USA; 3 Department of Molecular and Cellular Biology, City of Hope, Duarte, USA; 4 GeneDx, Gaithersburg, USA; 5 Corporate Executive Board (CEB), Arlington, USA; 6 National Center for Biotechnology Information, National Library of Medicine, National Institutes of Health, Bethesda, USA

**Keywords:** dbGaP, TCGA, SRA, cancer, database, genome, The Cancer Genome Atlas, GDC

## Abstract

Data sharing is critical to advance genomic research by reducing the demand to collect new data by reusing and combining existing data and by promoting reproducible research. The Cancer Genome Atlas (TCGA) is a popular resource for individual-level genotype-phenotype cancer related data. The Database of Genotypes and Phenotypes (dbGaP) contains many datasets similar to those in TCGA. We have created a software pipeline that will allow researchers to discover relevant genomic data from dbGaP, based on matching TCGA metadata. The resulting research provides an easy to use tool to connect these two data sources.

## Introduction

Many large funding organizations, including the National Institutes of Health (NIH), encourage researchers to make their data available in public databases. Policies like the NIH’s Genomic Data Sharing policy ( https://gds.nih.gov/03policy2.html) and other incentives around data sharing have promoted the development of several public data repositories. However, in spite of the availability of data, it can still be challenging to harness the power of these public databases, and researchers are faced with a variety of barriers in accessing shared data ( van Schaik *et al.*, 2014).

A major obstacle to data discovery is the disconnectedness of various data sharing resources. Automated tools that can connect these databases and reduce the time that researchers spend on data discovery are critically needed ( Dudley & Butte, 2008; Ruau *et al.*, 2011). Such tools will promote reproducibility, increase the efficiency of research, and aid in solving the problem of small sample sizes. These issues are especially relevant to genomic data, which is typically expensive to gather.

Here, we focus on connecting two popular genomic data repositories, the Database of Phenotypes and Genotypes (dbGaP) ( Tryka *et al*., 2014) and The Cancer Genome Atlas (TCGA), data hosted by the Genomic Data Commons (GDC; https://gdc.cancer.gov/). These two popular data sharing resources both house genomic datasets related to cancer, but despite containing similar data, these repositories have no direct connection to allow researchers to link them together. In the case of these two repositories the only way to find projects with analogous metadata is to manually search each repository. The key contribution of this work is a tool that acts as an interface between the GDC and dbGaP, which allows researchers to discover dbGaP datasets with similar metadata to a TCGA dataset of interest.

## Methods

### 1. Resources

***GDC.*** The GDC ( https://gdc.cancer.gov/) is a highly curated resource for datasets from cancer related genomic studies from the National Cancer Institute (NCI). Its primary function is to provide a centralized repository for accessibility to data from large-scale NCI programs, such as ***TCGA*** and its pediatric equivalent, Therapeutically Applicable Research to Generate Effective Treatments. As of September 2016, GDC held over 260K sequence files with different genomic data-types (whole genome, RNA, etc.) of over 14K patients.

***dbGaP.*** The National Center for Biotechnology Information (NCBI) dbGaP ( https://www.ncbi.nlm.nih.gov/gap) is the largest collection of genomic data. It is not limited to cancer data or human data. While the metadata fields are fixed, unlike the GDC, the entries in these fields are not curated. This is a challenge for harmonizing the metadata across the two datasets. The NCBI Sequence Read Archive (SRA) ( https://www.ncbi.nlm.nih.gov/sra) is a collection of sequence data associated with the studies in dbGaP.

### 2. Development

As the tool was developed as part of a hackathon, we used a development methodology similar to the Rapid Application Development model suitable for prototype development ( Kerr & Hunter, 1994). This subsection is organized as steps within this methodology.

***Defining the scope.*** We first identified the end users of our tool to be molecular and computational biologists and bioinformaticians with limited programming experience. Thus, the tools had to be easy to setup and execute. Next, we identified the use-cases as follows:

- The tool should take TCGA study identifiers or study-level metadata values from the GDC and identify dbGaP studies with analogous data.
- The tool should subsequently provide the capability of fetching the sequence level genomic data directly for these studies from the NCBI SRA data repository.

This gave us the necessary modules that needed to be developed.

***Mapping the metadata.*** We first extracted the required metadata by parsing the raw XML data and also scrapping the website data from both TCGA (GDC) and dbGaP. This metadata is stored as mapping tables in CSV format. Based on the extracted metadata, we developed two mapping dictionaries to translate between 1) disease terms and 2) genomic data-types, as defined separately within dbGaP and the GDC.

Accomplishing this mapping was challenging, as the allowable values for these fields is strictly controlled in the GDC, but completely user-defined in dbGaP. We designed a rule-based mapper to generate an initial map between search values from each repository, then manually curated these mappings to refine and rank mapped terms. These mappings are stored and used during the execution of our tool.

***Developing the required modules.*** Both the TCGA data (through GDC; https://gdc.cancer.gov/developers/gdc-application-programming-interface-api) and dbGaP (through NCBI Eutils; https://eutils.ncbi.nlm.nih.gov/entrez/eutils/) provide APIs to access their respective data that allow metadata transfer in the XML or JSON formats. An API or Application Programming Interface provide an interface to data and services that other programs can directly use.

The SRA toolkit is a software tool that allows researchers to obtain the sequence data (with appropriate access rights) from the SRA database. The search can be narrowed by various parameters, including the genomic region and type of sequence (e.g. mRNA and whole genome shotgun).

We used Python (version 2.7; https://www.python.org/) for the development of our tool. We wanted to keep the tool as platform agnostic as possible. As the SRA toolkit is Unix-based, only the final part of the implementation pipeline, as discussed subsequently, is a shell script (not directly compatible in Windows environment).

## Results

**Figure 1.**
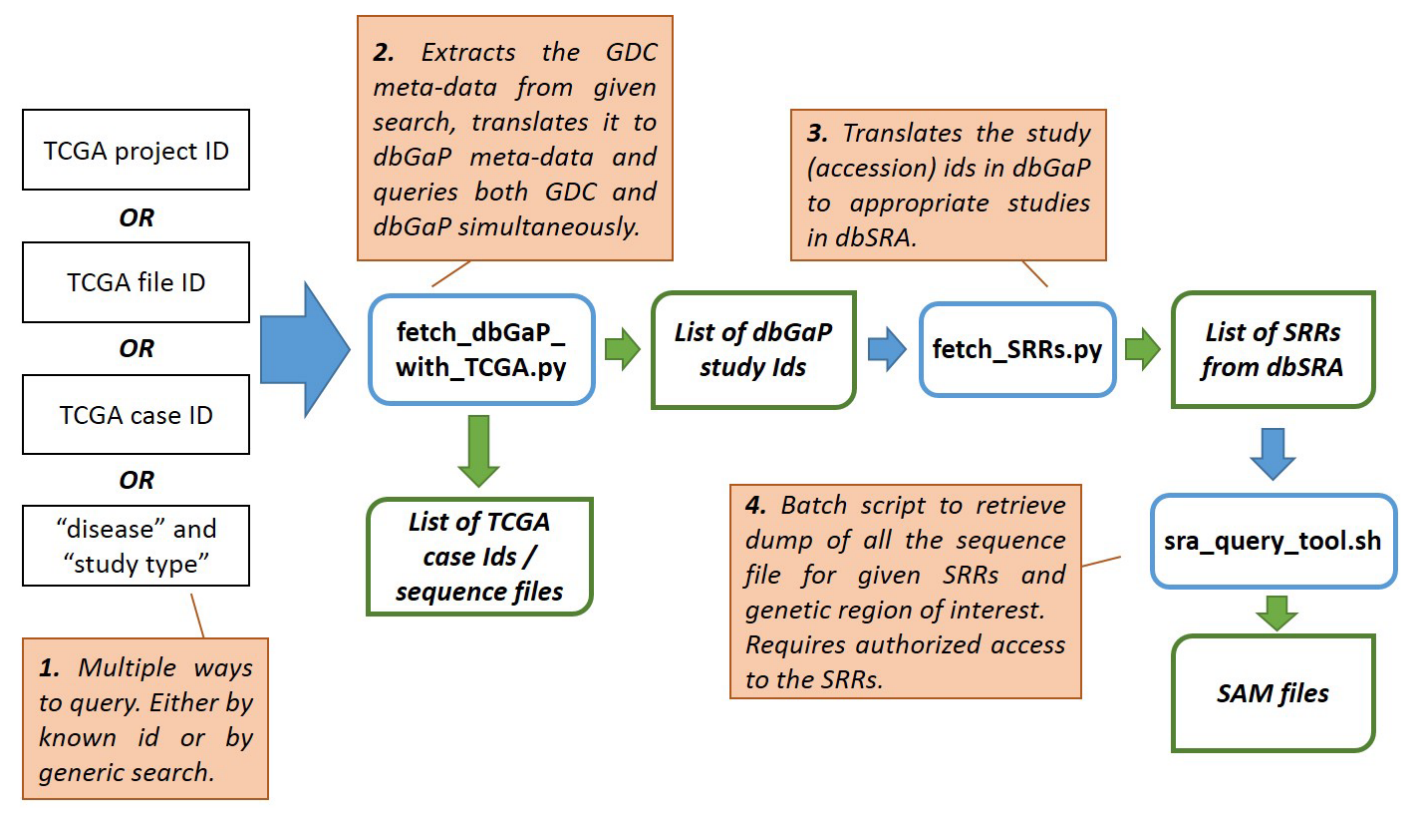
Module organization and a typical end-to-end workflow.

We developed an easy-to-use tool that can be used to find additional data from dbGaP (and SRA) by expanding TCGA queries automatically. The first part of the pipeline allows researchers to query either repository by TCGA Project ID, File ID, Case ID, disease type, or experimental strategy via a metadata mapping dictionary. It returns not only a list of TCGA IDs, but also a list of related dbGaP study IDs. For dbGaP studies with NCBI SRA data, the second part of the pipeline will return the .sam files that contains reads aligned to a genomic region of interest to be used with the SRA Toolkit. Our tool is divided into three modules as illustrated in Figure 1. Below, each module is discussed in detail.

### 1. Fetching dbGaP studies using TCGA data

This component of the pipeline queries the GDC in multiple ways, including a direct ID search for projects, cases, samples, or files, or a custom search by the cancer type or experimental methods. Currently, the scope of custom search is limited to the available terms in the GDC data portal ( Table 1). The module fetches the metadata using the GDC API and extracts the metadata terms related to the specified ID (i.e. the cancer type and experiment method). It then translates these terms to corresponding dbGaP search terms and returns the relevant dbGaP study IDs using the NCBI Eutils API. While executing the pipeline, the XML/JSON outputs of the APIs are processed in-memory behind the scenes. Thus, the end-users are not exposed to the API directly.

**Table 1.** List of allowable values for Study Type, Primary Site and Disease in the Genomic Data Commons (The Cancer Genome Atlas) data. The mapping between the Disease and Primary Site can be found in our GitHub repository.

Study Type		Primary Site		Disease
Genotyping Array		Adrenal Gland		Pheochromocytoma and Paraganglioma
miRNA-Seq		Bile Duct		Adrenocortical Carcinoma
RNA-Seq		Bladder		Cholangiocarcinoma
WXS (Whole Exome Sequencing)		Blood		Bladder Urothelial Carcinoma
		Bone		Acute Myeloid Leukemia
		Brain		Osteosarcoma
		Breast		Glioblastoma Multiforme
		Cervix		Brain Lower Grade Glioma
		Colorectal		Breast Invasive Carcinoma
		Esophagus		Cervical Squamous Cell Carcinoma and Endocervical Adenocarcinoma
		Eye		Colon Adenocarcinoma
		Head and Neck		Rectum Adenocarcinoma
		Kidney		Esophageal Carcinoma
		Liver		Uveal Melanoma
		Lung		Head and Neck Squamous Cell Carcinoma
		Lymph Nodes		High-Risk Wilms Tumor
		Nervous System		Kidney Renal Clear Cell Carcinoma
		Ovary		Kidney Renal Papillary Cell Carcinoma
		Pancreas		Kidney Chromophobe
		Pleura		Rhabdoid Tumor
		Prostate		Clear Cell Sarcoma of the Kidney
		Skin		Liver Hepatocellular Carcinoma
		Soft Tissue		Lung Adenocarcinoma
		Stomach		Lung Squamous Cell Carcinoma
		Testis		Lymphoid Neoplasm Diffuse Large B-cell Lymphoma
		Thymus		Neuroblastoma
		Thyroid		Ovarian Serous Cystadenocarcinoma
		Uterus		Pancreatic Adenocarcinoma
				Mesothelioma
				Prostate Adenocarcinoma
				Skin Cutaneous Melanoma
				Sarcoma
				Stomach Adenocarcinoma
				Testicular Germ Cell Tumors
				Thymoma
				Thyroid Carcinoma
				Uterine Corpus Endometrial Carcinoma
				Uterine Carcinosarcoma

For custom searches, this module returns results from both the GDC and dbGaP simultaneously. Thus, this module also provides consolidated search capability over the TCGA and dbGaP data. The output from this module includes two files:

- a list of the TCGA cases for the given project or search criteria, and
- a list of dbGaP studies (with links) that are analogous to the input query.

### 2. Fetch SRRs for given dbGaP studies

The second component of the pipeline takes the list of dbGaP studies IDs and returns the list of sequence read run (SRR) files from the NCBI SRA from the dbGaP studies, when available. The users can specify the genomic region of interest as an additional parameter.

### 3. Fetch the sequence files from SRRs

The final part of the pipeline takes a list of SRRs and uses the SRA-toolkit to return sequencing level genomic data for a genomic region of interest directly from NCBI SRA data repository. This module assumes the required authorization has been granted prior to accessing the sequencing data.

## Conclusion

To our knowledge, this is the first easy-to-use tool for harmonizing TCGA and dbGaP study metadata for the purpose of data discovery and consolidated querying. We would like to continue to work with the cancer biology community to develop this interface tool. Future improvements include extending our search capabilities to include other metadata, the option to query multiple genomic regions simultaneously, and a user-friendly GUI. Feature requests or contributions of code can be made on our GitHub site, which will be monitored for such activity.

## Software availability

Latest source code: https://github.com/NCBI-Hackathons/TCGA_dbGaP.

Archive source code as at the time of publication: doi, 10.5281/zenodo.160551 (Kurata, 2016) ( https://zenodo.org/record/160551#.WE7Lz9WLTcs)

License: CC0 1.0 Universal
