# Supplementary material for: Extending TCGA queries to automatically identify analogous genomic data from dbGaP
Source: F1000Res. 2017 Mar 24;6:319. [Version 1] doi: 10.12688/f1000research.9837.1 (PMC5538035; doi:10.12688/f1000research.9837.1)
Supplement: Peer review history

# Peer review: Referee response for version 1

**DOI:** 10.5256/f1000research.10605.r23937

**Published:** 2017-07-28

**Authors:** Tsung-Jung Wu

**Affiliations:** 1 Baylor College of Medicine, Houston, TX, USA

The software can fulfill the requirement of authors designated task. Since the original design of this software is not for general usage, it might be difficult for a general user to access. However, with this tools' help, a cancer genomics researcher can download database from both GDC and dbGAP sample info easily. The most important part of this software is manually curated terms. This approach can assure more accurate search outcome and save user's valuable time. By introducing Disease Ontology to the mapping between the Disease and Primary Site step, it might be able to help more accurate curation and cancer type determination. Articles provide here are about Disease Ontology and Disease Ontology Cancer Slim. The tool is designed for cancer genomics community and with these terminologies and ID. This tool will be able to further expand its usage and application.

 Some more documentations of this software and outcome interpretations will be helpful.

I have read this submission. I believe that I have an appropriate level of expertise to confirm that it is of an acceptable scientific standard.

## References

[1] KibbeWAArzeCFelixVMitrakaEBoltonEFuGMungallCJBinderJXMaloneJVasantDParkinsonHSchrimlLM: Disease Ontology 2015 update: an expanded and updated database of human diseases for linking biomedical knowledge through disease data. *Nucleic Acids Res*.2015;43(Database issue) : 10.1093/nar/gku1011 D1071-8 10.1093/nar/gku1011 25348409PMC4383880

[2] WuTJSchrimlLMChenQRColbertMCrichtonDJFinneyRHuYKibbeWAKincaidHMeerzamanDMitrakaEPanYSmithKMSrivastavaSWardSYanCMazumderR: Generating a focused view of disease ontology cancer terms for pan-cancer data integration and analysis. *Database (Oxford)*.2015;2015: 10.1093/database/bav032 bav032 10.1093/database/bav032 25841438PMC4385274

# Peer review: Referee response for version 1

**DOI:** 10.5256/f1000research.10605.r23935

**Published:** 2017-07-10

**Authors:** Konstantinos Krampis

**Affiliations:** 1 Department of Biological Sciences, Hunter College of The City University of New York, New York, NY, USA

The software is adequately explained and is a useful tool for a specialized task, fitting the format and section of the F1000. It is great work given that this was all completed during the hackathon. However I would suggest some polishing of the readme on the Github. This will still not make it any easier to use for not expert users, but for this purpose it would be ideal if the authors can add a short software readme as supplementary to the manuscript. The citation I have provided with this review points to a paper for the BioDocklets software that includes a manual that the authors could use as example. Other than that this is a great article that provides a very useful tool integrating key aspects of two important databases for the community.

I have read this submission. I believe that I have an appropriate level of expertise to confirm that it is of an acceptable scientific standard.

## References

[1] KimBAliTLijeronCAfganEKrampisK: Bio-Docklets: virtualization containers for single-step execution of NGS pipelines. *GigaScience*.2017; 10.1093/gigascience/gix048 10.1093/gigascience/gix048 PMC556992028854616

# Peer review: Referee response for version 1

**DOI:** 10.5256/f1000research.10605.r21260

**Published:** 2017-04-28

**Authors:** Yussanne Ma, Victoria Trinh

**Affiliations:** 1 Canada's Michael Smith Genome Sciences Centre, BC Cancer Agency, Vancouver, BC, Canada

The authors present a clear description of a tool that is simple in concept but will be of use to the cancer genomics community. They identified a clear need for researchers to be able to easily identify and download datasets from GDC and dbGAP sample attributes and have designed a solution that is conceptually sound. Most helpfully, they have taken care of not only the manual search but the mapping of the non-homogeneous metadata fields to save users time.

 My detailed comments are listed below:

 1. (Major) After installing the code and running the first command, we received this error:

 $ bin/python bin/fetch_dbGaP_with_TCGA.py id -i TCGA-BRCA -s project -l low Traceback (most recent call last):

   File "bin/fetch_dbGaP_with_TCGA.py", line 332, in

     sys.exit(main())

   File "bin/fetch_dbGaP_with_TCGA.py", line 322, in main

    

 +outDict[outStringKeys[returnType][2]]+","+"\\".join(outDict[outStringKe

 +ys[

 returnType][3]])+","+outDict[outStringKeys[returnType][4]]+"\n"

 TypeError: coercing to Unicode: need string or buffer, list found

 As the authors are targeting users with minimal coding experience, it is important that error messages are far more informative. From the above it's completely unclear without looking into the code itself whether this is due to the input being in an incorrect format or if there is an actual problem with the code itself. Much more user testing and error handling is needed.

 2. (Major) In general, more user documentation is needed. The instructions are brief and again, do not account for the case of everything not working on the first try. Input examples and example commands should also be provided for fetch_dbGaP_with_TCGA.py. Notes on how to interpret the output would also be helpful, perhaps by annotating the output file examples provided, which are a good inclusion.

 3. (Minor) Currently the only way to see the re-mapping of metadata is to look in the code itself on github. Could a txt file of the field mappings be provided? This would be helpful for users to understand the assumptions being made with the metadata vocabulary.

 4. (Minor) In the results section, could the authors summarize the results of the testing they did to ensure that the results being returned are correct? At minimum, it is important that users are assured of the completeness of the search. This could, for example, be demonstrated by searching on a TCGA disease type in GDC and comparing the number of results with the TCGA cohort size. Specificity and accuracy of the search results should also be demonstrated, perhaps by showing and summarizing the results of some example searches in both sites.

 5. (Minor) GDC currently hosts not only TCGA, but also TARGET data (which is mentioned in the manuscript), and will soon be hosting other datasets. Is this tool limited to the TCGA datasets in GDC? As far as I know from our submissions to the GDC, the other datasets will have the same controlled metadata fields so the functionality should extend naturally to all of the data hosted at the GDC and it would greatly increase the utility if this were the case.

 I think this tool will be beneficial to the cancer genomics community and will facilitate and encourage users to mine the rich NGS datasets that have been made available in the past decade. It would be good if the authors are able to provide a revision of the code that works and make some improvements to the user documentation, so that these benefits can be realized.

We have read this submission. We believe that we have an appropriate level of expertise to confirm that it is of an acceptable scientific standard, however we have significant reservations, as outlined above.
